# A nanobody:GFP bacterial platform that enables functional enzyme display and easy quantification of display capacity

**DOI:** 10.1186/s12934-016-0474-y

**Published:** 2016-05-03

**Authors:** Sofie Wendel, Emil C. Fischer, Virginia Martínez, Susanna Seppälä, Morten H. H. Nørholm

**Affiliations:** Novo Nordisk Foundation Center for Biosustainability, Technical University of Denmark, Kogle Allé 6, 2970 Hørsholm, Denmark

**Keywords:** Surface display, Nanobody, GFP, Chitinase A, LppOmpA, Autotransporter, Whole-cell catalysis

## Abstract

**Background:**

Bacterial surface display is an attractive technique for the production of cell-anchored, functional proteins and engineering of whole-cell catalysts. Although various outer membrane proteins have been used for surface display, an easy and versatile high-throughput-compatible assay for evaluating and developing surface display systems is missing.

**Results:**

Using a single domain antibody (also called nanobody) with high affinity for green fluorescent protein (GFP), we constructed a system that allows for fast, fluorescence-based detection of displayed proteins. The outer membrane hybrid protein LppOmpA and the autotransporter C-IgAP exposed the nanobody on the surface of *Escherichia coli* with very different efficiency. Both anchors were capable of functionally displaying the enzyme Chitinase A as a fusion with the nanobody, and this considerably increased expression levels compared to displaying the nanobody alone. We used flow cytometry to analyse display capability on single-cell versus population level and found that the signal peptide of the anchor has great effect on display efficiency.

**Conclusions:**

We have developed an inexpensive and easy read-out assay for surface display using nanobody:GFP interactions. The assay is compatible with the most common fluorescence detection methods, including multi-well plate whole-cell fluorescence detection, SDS-PAGE in-gel fluorescence, microscopy and flow cytometry. We anticipate that the platform will facilitate future in-depth studies on the mechanism of protein transport to the surface of living cells, as well as the optimisation of applications in industrial biotech.

**Electronic supplementary material:**

The online version of this article (doi:10.1186/s12934-016-0474-y) contains supplementary material, which is available to authorized users.

## Background

Cell factories are a promising alternative to the problematic fossil fuel-based technologies currently employed in industry [1]. Cellular surface display of proteins is an attractive way to engineer whole-cell catalysts, thereby reducing time, cost and effort related to enzyme purification and one-time use of enzyme batches. Displaying proteins on the cell surface has the evident benefits of omitting any need to transport substrate or product across the cell wall, and may reduce toxicity effects due to the extracellular location of pathway components, substrates and products. The first successful cases of surface display were reported more than three decades ago [2, 3] but as pointed out by Schüürmann et al. [4] industrial development of whole-cell catalysts is lagging behind. Surface display has been successfully carried out on several platforms such as yeast [5], phage [6] and bacteria [4] with several different cargos (typically antibodies or enzymes). Nevertheless, detailed understanding of the molecular mechanisms underlying surface display is lacking, thus complicating rational design [4, 7–9]. Furthermore, the development of display systems suffers from a lack of simple and wide-ranging assay methods, which would allow for easy detection and optimisation. Recently, an increasing number of studies have shown the use of fluorescently labelled antibodies as a non-generic method for visualising surface displayed proteins, and enabling not only their detection but also flow cytometric analysis and microscopy [10–12].

**Fig. 1:**
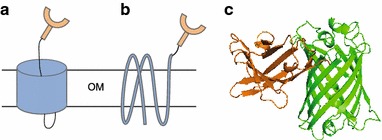
Illustrations of the nanobody:GFP complex and the outer membrane anchors. **a**, **b** Schematic illustration of the nanobody (*orange*) **a** as passenger of the autotransporter C-IgAP construct and **b** fused to the outer membrane protein OmpA. **c** Crystal structure of the enhancer nanobody binding GFP (PDB ID: 3OGO; [32]). OM outer membrane

Several different membrane anchors have been explored for bacterial surface display; two of the main ones are autotransporters and outer membrane proteins from gram-negative bacteria (for review see e.g. [4, 13]). Autotransporters are typically involved in virulence and have been extensively explored for bacterial surface display [14–16]. Autotransporter proteins consist of an N-terminal signal peptide that directs the polypeptide through the plasma membrane, a passenger domain that typically encodes a virulence factor, and a C-terminal translocation unit that enables the transport of the passenger domain across the outer membrane (Fig. 1a) [9]. While the transport of the passenger domain has given autotransporters their name, the whole protein as such is dependent on the barrel assembly machinery (BAM) complex to reach the cell surface [17, 18]. The C-terminal translocation unit of the *Neisseria gonorrhoeae* autotransporter IgA protease (C-IgAP) has been extensively characterised in terms of its mechanism of protein secretion as well as employed for surface display in *Escherichia coli* [19, 20]. Native *E. coli* outer membrane proteins constitute a different class of surface display anchors. The LppOmpA fusion, consisting of the Lpp signal peptide followed by five transmembrane segments of Outer membrane protein A, has been successfully used to display enzymes such as hydrolases on the surface of *E. coli* (Fig. 1b) [11, 21].

Surface display of proteins has many parallels to bacterial membrane protein production, which is an inherently difficult process, dependent on proper balancing of the transport machineries and with optimal process conditions varying for different proteins [22–24]. The detection and optimisation of membrane protein production was dramatically simplified by the development of a green fluorescent protein (GFP)-fusion platform that enabled real-time monitoring, quantification and fast analysis of protein integrity and membrane association [25, 26]. As pointed out by Sun et al. [10] displaying GFP on the surface of cells could similarly be used to assess surface display levels. This approach, however, comes with a major drawback: cells producing folded GFP would be fluorescent regardless of whether the GFP protein was actually displayed on the cell surface or not, i.e. if the protein remained in the cytoplasm or periplasm. Only with methods like advanced microscopy or complicated separation of compartments could one differentiate between surface localised and cyto/periplasmic GFP signal, and no conclusions about display efficiency could be drawn from a simple readout like whole-cell fluorescence. We therefore set out to develop a fluorescence-based method for surface display evaluation using an alternative approach, making use of a single-chain antibody molecule known as a nanobody.

Nanobodies (NB), found in camelids and sharks, are single domain antibodies that carry out the same function as full-size antibodies whilst consisting of only a variable heavy fragment [27, 28]. The small size of nanobodies makes them convenient protein tags, and the absence of essential disulphide bonds makes them easy to produce in *E. coli* [29, 30]. Kirchhofer et al. developed nanobodies that bind GFP with high specificity and affinity in a stable complex; in fact, the complex is stable enough to sustain denaturing SDS-PAGE analysis (Fig. 1c) [31, 32]. Here, we have constructed a system for fluorescence-based detection of surface display by fusing the GFP-nanobody to different outer membrane anchors and visualising the displayed protein by adding purified GFP to whole cells.

## Results

### Construction of nanobody modules for surface display

**Fig. 2:**
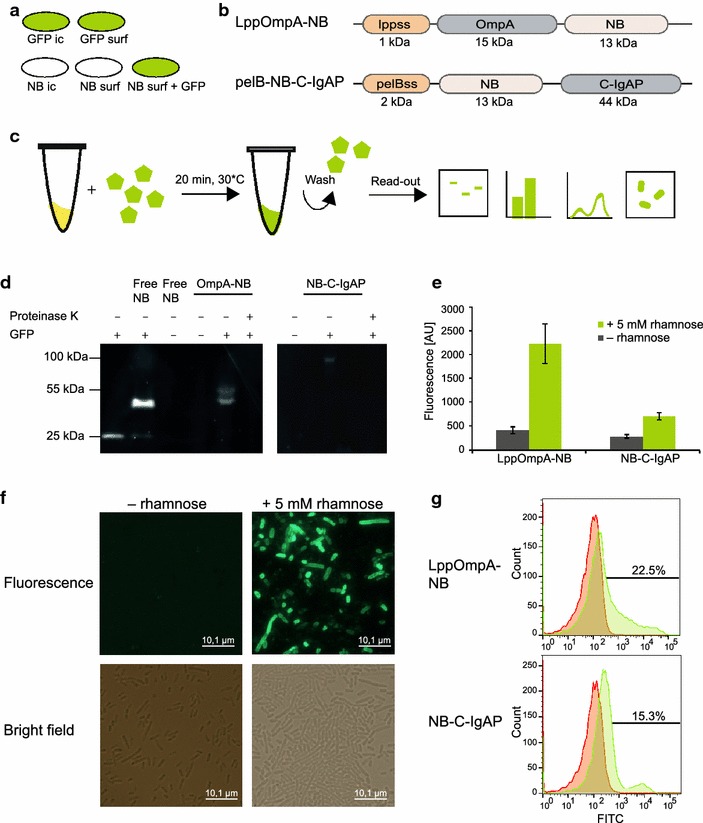
Characterisation of the NB:GFP platform. **a** Illustration of the principal difference between displaying GFP and displaying the nanobody on the surface of the cell. GFP gives the cell a fluorescent glow whether produced intracellularly (ic) or on the surface (surf). In contrast, the only way a cell producing the nanobody can be fluorescent is if the nanobody is displayed on the surface and accessible to extracellular GFP. **b** Protein schemes for the OmpA and autotransporter constructs. An N-terminal signal sequence (lppss and pelBss) precedes the OmpA anchor followed by the nanobody, or the nanobody followed by the C-IgAP anchor, respectively. **c** Workflow of GFP assay: cells producing the nanobody are incubated with free GFP; unbound GFP is washed off and the fluorescence signal from GFP bound to nanobody on cells is assayed using SDS-PAGE, fluorescence measurements and microscopy. **d** In-gel fluorescence of purified GFP; purified GFP mixed with purified NB; NB; whole cells displaying OmpA-NB or NB-C-IgAP with and without GFP and with and without proteinase K treatment. The same amount of cells was loaded in* each lane* for whole-cell samples. **e** Whole-cell fluorescence measurement with and without rhamnose induction. Values are averages of three biological replicates and* bars* show standard error. **f** Bright field and fluorescence microscopy images of OmpA-NB displayed on *E. coli* cells, with and without rhamnose induction. **g** Flow cytometry profiles of pK:LppOmpA-NB and pK:NB-C-IgAP with induction (*green*) and without induction (*red*). Percentage numbers show the fraction of cells that are fluorescent. *Lppss* lpp signal sequence, *pelBss* pelB signal sequence, *OmpA* Outer membrane protein A, *NB* nanobody, *C-IgAP* C-terminal of IgA protease

GFP as reporter for surface displayed proteins is problematic, because it is difficult to differentiate between intracellular and surface displayed protein. Therefore, we used a complementary approach where the surface displayed protein is fused to a GFP-nanobody and subsequently detected using purified GFP added from the outside (Fig. 2a).

Two different display modules containing the nanobody were constructed, using the previously described GFP-enhancer-nanobody sequence [31]. As anchors, we chose two commonly used outer membrane proteins: We designed one display vector containing an Outer membrane protein A (OmpA) domain, and one vector containing an autotransporter domain, in both cases using the high-copy plasmid pKS1, herein called pK [33]. The outer membrane protein-based vector pK:LppOmpA-NB contains the N-terminal signal peptide of the *E. coli lpp* gene (residues M1–Q29), followed by residues N66–G180 of OmpA (forming five beta-strand transmembrane segments) and a C-terminally-fused nanobody sequence (Fig. 2b). An alternative vector, pK:pelB-NB-C-IgAP, was constructed by fusing the nanobody in-between the pelB signal peptide and the C-terminal domain of the *N. gonorrhoeae* autotransporter IgA protease (C-IgAP) (Fig. 2b). In both cases, protein production is under the control of the rhamnose-inducible *rhaP*_*BAD*_ promoter.

### Functional, surface displayed nanobody is robustly assayed using GFP

pK:LppOmpA-NB and pK:NB-C-IgAP were transformed into *E. coli* BL21(DE3) and protein production was induced in liquid culture by the addition of 5 mM rhamnose. After 3 h of induction, cells were harvested, resuspended in buffer and incubated with purified GFP for 20 min at 30 °C. Cells were harvested and washed twice with buffer to remove any unbound GFP; the repeated centrifugation steps also ensured that only whole cells were assayed. The washed cells were then subjected to (1) plate reader fluorescence measurement, (2) SDS-PAGE and in-gel fluorescence analysis, (3) flow cytometry analysis and (4) fluorescence microscopy (Fig. 2c). In all cases we could detect a fluorescence signal, showing the versatility of the nanobody:GFP platform (Fig. 2d–g).

Both the autotransporter C-IgAP and LppOmpA anchors successfully displayed the nanobody, as confirmed by in-gel fluorescence of OD-normalised whole-cell samples after incubation with GFP (Fig. 2d). The very fact that cells are fluorescing shows that the nanobody is accessible from the outside of the cell, and surface localisation is further confirmed by Proteinase K assay, removing all signal (Fig. 2d). The GFP signal is confined to bands corresponding to a complex of GFP bound to the NB construct (theoretical sizes 55 and 85 kDa, respectively) and none of the fluorescence appear to originate from free GFP (27 kDa, Fig. 2d). Whole-cell fluorescence was measured in a plate reader and used to evaluate and quantify display ability of the entire bacterial population (Fig. 2e). Induced cultures were highly fluorescent compared to uninduced cultures, and the cultures containing the LppOmpA anchor showed approximately three times higher fluorescence than the C-IgAP cultures. Nanobody-displaying cells were also visualised using fluorescence microscopy: uninduced cells incubated with GFP and then washed prior to microscopy showed no fluorescence signal, while induced cells were strongly fluorescent (Fig. 2f). Single-cell display behaviour was analysed by flow cytometry, which revealed that a fraction of the cells (22.5 % for LppOmpA and 15.3 % for C-IgAP) were responsible for the majority of the fluorescence (Fig. 2g). Also, the top LppOmpA expressers reached a fivefold higher fluorescence value than the corresponding C-IgAP cells. C-IgAP producing cells were much more negatively affected by induction than LppOmpA cells; OD dropped by 70 % when inducing NB-C-IgAP, and this was not affected by varying inducer concentration (Additional file 1: Figure S1). In contrast, the density of LppOmpA cultures decreased gradually and less dramatically upon induction (Additional file 1: Figure S1). Background fluorescence from the added GFP was essentially absent, as seen by whole cell fluorescence for uninduced cells, in-gel fluorescence, microscopy, and flow cytometry (Fig. 2e–g). The detection system was functional and robust in both small and large format, with 96-well format allowing high-throughput analyses.

### Displaying a functional enzyme as GFP-detectable nanobody fusions

**Fig. 3:**
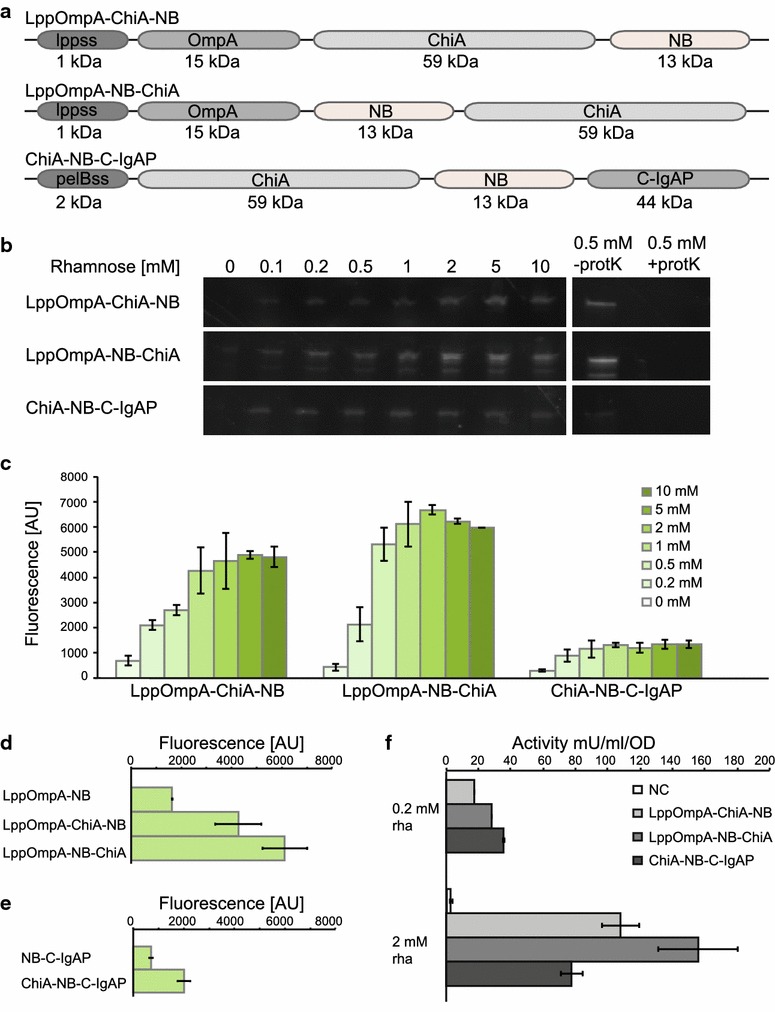
Functional display of Chitinase A using both display anchors. **a** Protein schemes for Chitinase A-NB fusions. The ChiA protein was fused either in-between OmpA and NB, or C-terminally to LppOmpA-NB. With the C-IgAP anchor, ChiA was fused N-terminally to NB-C-IgAP. **b** In-gel fluorescence of rhamnose titrations of chitinase-nanobody fusions, and in-gel fluorescence after addition of proteinase K. The same amount of cells was loaded in* each lane*. **c** Whole-cell fluorescence for rhamnose titration of each of the chitinase-nanobody fusions. Values are averages of biological duplicates, *error bars* are standard errors. **d** Whole cell fluorescence for LppOmpA constructs with and without ChiA, induced with 1 mM rhamnose. Values are averages of biological duplicates, *bars* show standard error. **e** Whole cell fluorescence for C-IgAP constructs with and without ChiA, induced with 1 mM rhamnose. Values are averages of biological triplicates, *bars* show standard error. **f** Specific chitinase activity for nanobody-chitinase fusions at two different inducer concentrations, normalised to OD. Values are averages of biological duplicates, *bars* are standard errors. *Lppss* lpp signal sequence, *pelBss* pelB signal sequence, *OmpA* Outer membrane protein A, *ChiA* Chitinase A, *NB* nanobody, *C-IgAP* C-terminal of IgA protease

The application of the nanobody platform was further tested by making sandwich fusions to the Chitinase A enzyme from *Serratia marcescens* (courtesy of Prof. Victor de Lorenzo, CNB, Madrid). ChiA is an industrially relevant enzyme for biotechnology applications [34]. The chitinase was fused either N- or C-terminally to the nanobody in the pK:LppOmpA-NB construct, and N-terminally to the nanobody in the pK:NB-C-IgAP construct, resulting in a total passenger size of 72 kDa (Fig. 3a). The proteins were subsequently produced in *E. coli* BL21 (DE3) and surface exposure was assayed using GFP as described above. Successful display was confirmed by in-gel fluorescence (Fig. 3b), and whole-cell fluorescence (Fig. 3c). This demonstrated that the nanobody was fully functional and binding its antigen GFP also when fused to another, large protein, and even when sandwiched in-between two proteins. As for the initial nanobody constructs, significant differences in display efficiency were observed as an effect of anchor usage. Interestingly, fusing the chitinase to the nanobody considerably increased display efficiency for both the LppOmpA-anchor and C-IgAP (Fig. 3d, e). Furthermore, the position of the nanobody in the construct influenced the surface presentation; pK:LppOmpA-NB-ChiA showed substantially higher fluorescence than pK:LppOmpA-ChiA-NB.

The rhamnose promoter is highly titratable [35, 36]. To test this tunability in our system, we varied inducer concentration from 0 to 10 mM rhamnose (Fig. 3b, c). Based on in-gel fluorescence analysis and plate reader data, increasing the concentration of rhamnose led to higher protein production, but the effect levelled off at higher concentrations. The LppOmpA constructs showed better tunability by rhamnose than the autotransporter version, which was largely unaffected by inducer concentration.

To confirm the functionality of the surface displayed enzyme, chitinolytic activity was assayed in vivo, for the same amount of cells, at two different inducer concentrations, 0.2 and 2 mM rhamnose (Fig. 3f). The chitinase was active with all surface display constructs, with higher activity for the LppOmpA fusions pK:LppOmpA-NB-ChiA (156 ± 25 mU/ml/OD at 2 mM rhamnose induction) and pK:LppOmpA-ChiA-NB (108 ± 11 mU/ml/OD) than for the autotransporter variant pK:ChiA-NB-C-IgAP (77 ± 7 mU/ml/OD). Activity levels correlated well with GFP-based expression data measured with plate reader and in-gel fluorescence for the LppOmpA fusions, with a doubling of fluorescence corresponding to a doubling in activity (Fig. 3c, f). This correspondence between enzymatic activity and fluorescence data showed that the NB:GFP assay gives a reliable indication of how much functional protein is displayed on the cell surface for LppOmpA. For pK:ChiA-NB-C-IgAP the correlation is weaker, with activity levels varying more than fluorescence levels for the two inducer concentrations. Controls without chitinase showed no background activity.

### Flow cytometry analysis reveals two populations of cells and confirms varying display efficiency

**Fig. 4:**
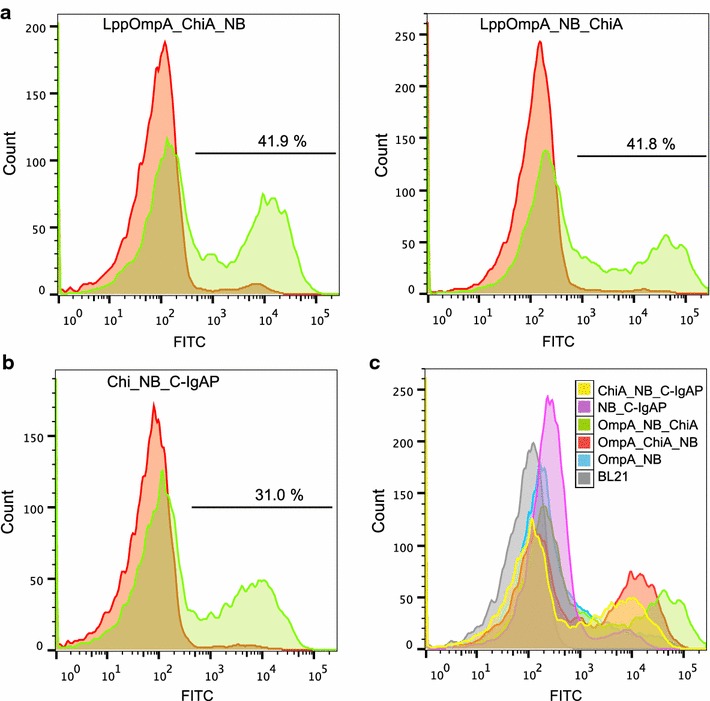
FACS-analysis of Chitinase A-nanobody surface display fusions. Fluorescence was measured by flow cytometry 3 h after rhamnose induction of cells in exponential growth followed by 20 min incubation with purified GFP, and two steps of washing. **a** Chitinase-nanobody fusions surface displayed with LppOmpA, with (*green*) and without (*red*) rhamnose induction. **b** Chitinase-nanobody fusion displayed with C-IgAP, with (*green*) and without (*red*) rhamnose induction. **c** Overlays of the flow cytometry profiles for all fusions, with rhamnose induction: LppOmpA-NB, LppOmpA-ChiA-NB, LppOmpA-NB-ChiA, NB-C-IgAP, and ChiA-NB-C-IgAP

To study the display efficiency on a single-cell level, cells were analysed by flow cytometry (Figs. 2g, 4). This revealed two disparate populations of cells, both when using the LppOmpA anchor and the C-IgAP anchor (Fig. 4a, b, respectively), with only one of the populations presenting the protein fusion on the cell surface, as previously observed [10, 37]. The proportion of fluorescent cells varied from only 15.3 % for pK:NB-C-IgAP to 41.9 % for pK:LppOmpA-ChiA-NB (Fig. 4c). Interestingly, the relative amount of displayers was virtually identical for pK:LppOmpA-ChiA-NB (41.9 %) and pK:LppOmpA-NB-ChiA (41.8 %), but the mean fluorescence as measured by flow cytometry is 2.5-fold higher for pK:LppOmpA-NB-ChiA, in line with whole-cell fluorescence (Fig. 3c). Thus, the pK:LppOmpA-NB-ChiA population contained cells that had very high fluorescence per cell, while the fluorescent population was more homogeneous in the case of pK:LppOmpA-ChiA-NB. This highlights the different levels of information obtained from the different methods, and that high protein titers not necessarily correspond to high production per cell. The strikingly positive effect of Chitinase A on protein display levels was also evident with flow cytometry, with the proportion of fluorescent cells increasing by up to 86 % when adding Chitinase A to the LppOmpA fusion (compare Figs. 2g, 4a), and by 103 % for the corresponding C-IgAP fusion proteins (compare Figs. 2g, 4b). Under control of the *P*_trc_ promoter, the LppOmpA-NB construct formed a single population (Additional file 2: Figure S2).

### Nanobody:GFP platform allows systematic study of signal peptide effects on surface display

**Fig. 5:**
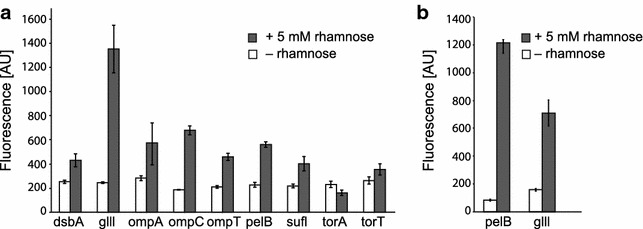
Evaluation of a signal sequence library on display levels of nanobody-fused chitinase (ChiA-NB-C-IgAP). **a** The ChiA-NB-C-IgAP fusion was cloned into a set of nine vectors containing different signal peptides for directing the polypeptide to the periplasm. Fluorescence values were measured in a plate reader. Significant variation among the different peptides was observed, with gIII showing the highest signal. Values are averages of biological triplicates, *bars* standard error. **b** Comparison of gIII and pelB signal peptides in the pK backbone. Values are averages of four biological replicates and* bars* show the standard error

With the assay running in a convenient 96-well format, it is possible to study how different parameters affect surface display in a high-throughput manner. As proof of concept, we studied the effect of the signal sequence on display efficiency. The challenging-to-display ChiA-NB-C-IgAP fusion was cloned into a set of nine low-copy pD881 plasmids, identical except for harbouring different signal peptides responsible for targeting the protein to the periplasm. We subsequently surveyed protein display using the NB:GFP assay, and compared whole-cell fluorescence values for the different constructs. Large variation was observed between the different signal sequences (Fig. 5a). Several of the signal peptides showed very low display capacity, in particular torA for which induced cells were negligibly fluorescent. The signal peptides dsbA, ompA, ompC, ompT, pelB, sufl and torT gave higher fluorescence values, whereas glll resulted in approximately twice as high fluorescence as the other signal peptides. Notably, pelB is the signal peptide used in the original pK construct, but, based on this result, it is only mediocre in comparison with glll. Therefore we followed up on this experiment by replacing the pelB signal peptide in the pK backbone with the gIII signal peptide and analysed protein production. In this high copy vector, the advantage of gIII over pelB disappeared (Fig. 5b). A signal sequence library was likewise constructed for the LppOmpA-ChiA-NB fusion. Again, the various signal sequences resulted in variation of enzyme display but without any exceptional high-displayer (Additional file 3: Figure S3).

## Discussion

Bacterial surface display of enzymatically active proteins is a promising strategy to engineer whole-cell catalysts, enabling simplification of production procedures as well as downstream processes. Even though many surface display systems have been successfully employed since the first bacterial anchors were developed in the 1980s (reviewed in [4]), unpredictable, cargo-dependent effects hinder rational design and optimization, as exemplified by Nicolay et al. [8] who reported failure to display a number of passenger domains with a previously characterised autotransporter.

In this study we used a camelid-derived nanobody to construct a quantitative, inexpensive, and robust assay that allows for easy GFP-based screening and optimization of surface display systems in a high-throughput manner. By making use of the tight association between a surface-presented nanobody and externally applied GFP, we addressed a need for a fluorescence-based assay for surface display, underscored by the avid use of two-step antibody labelling procedures in the literature [10–12]. Furthermore, we demonstrate that the NB:GFP platform is compatible with all techniques commonly associated with fluorescent tags: whole cell fluorescence measurements, in-gel fluorescence analysis, flow cytometry, and microscopy. Previously established techniques for fluorescence-based evaluation of surface display include the use of small peptide tags, such as FLAG and myc [38], and of domains of staphylococcal protein A and streptococcal protein G [39], followed by detection with antibodies. In relation to these, the NB:GFP platform differs especially in that it is a one-step procedure enabling all down-stream analyses, in particular simplifying analysis of samples on protein gels. Furthermore, costs for especially monoclonal antibodies are high, whereas both NB and GFP are easily produced in *E. coli*.

Both of the two anchors used, LppOmpA and C-IgAP, enabled functional display of the nanobody, although the efficiency was higher with LppOmpA in terms of total protein production as well as display efficiency across a population of cells. The basis for the difference in fluorescence could alternatively be low expression of the C-IgAP construct gene, poor folding of the nanobody, or poor GFP-NB interaction at the surface. The observed fitness cost for cells induced for NB-C-IgAP production, however, indicates that the explanation is meagre protein display, and points to LppOmpA as a more robust anchor in our system. While autotransporters have been reported as successful display anchors in many cases, our results are consistent with several studies where negative effects of autotransporter anchors were observed, with regards to cell viability [40], membrane integrity [41] and ultimately on surface display efficacy [8].

Although nanobodies are pharmaceutically interesting display targets in their own right (recently displayed in Gram positives [42] and *E. coli* [43], and routinely selected through phage-display [44]), our goal was to use the nanobody as a molecular biology tool for detection of other passenger-protein fusions. We applied the NB:GFP platform for surface display of the industrially relevant enzyme Chitinase A from *S. marcescens* [45, 46]. Its substrate chitin is one of the most abundant biomasses on Earth and degradation of chitin is attractive for e.g. bioethanol production, production of new materials and in the food industry [34].

Importantly, the nanobody was readily binding GFP independent of its position in the fusion protein: when displayed immediately linked to outer membrane proteins close to the cell surface; when being placed further away from the surface as fusions to the functional Chitinase A enzyme domain; and when being sandwiched in between the cell anchor and Chitinase A. This suggests that the nanobody is a robust fusion partner, also suitable for more complex designs such as multi-enzyme pathways. While GFP itself can be displayed on the cell surface [10, 47, 48], the complementary approach of displaying the nanobody and detecting it with externally added GFP circumvents the problem of false positives, since only binding with nanobody presented at the cell surface will yield a fluorescent signal.

Interestingly, adding Chitinase A to our protein fusions markedly enhanced display levels in spite of more than doubling the size of the fusion protein. A possible alternative explanation of the observed increase in fluorescence is that the nanobody became more accessible to GFP when fused to ChiA—this, however, seems unlikely given that the nanobody is sandwiched in-between two proteins in the best performing constructs. The apparent correlation between chitinolytic activity and GFP signal further supports an actual increased display level. Interactions between a passenger domain and the cell membrane have been suggested to be an important factor for translocation, and one could speculate that ChiA similarly stimulates translocation of its fusion partner [49]. In general, saturation of the transport machinery responsible for translocating proteins to the cell surface is a major bottleneck when overexpressing membrane protein genes [22]. Since Chitinase A is naturally secreted in its native gram-negative host, in contrast to the nanobody, it is likely that the biochemical properties of the protein are well suited for translocation in *E. coli* as well. The chitinase was active in all fusion combinations, and activity levels corresponded well with fluorescence data for LppOmpA, whereas correlation was weaker for the autotransporter version. This may be explained by some of the protein being halted in the periplasm, where the assay substrate might be accessible. This is in line with the observed fitness cost of NB-C-IgAP production. Although varying inducer concentrations made it possible to tune display to a certain degree, the increase in display eventually levelled off for the LppOmpA fusions, and C-IgAP display was only marginally affected. This fits well with previous reports that translocation is a generally limiting step in surface display [22, 47, 48].

The versatility of the NB:GFP platform allowed us to study the population behaviour by flow cytometric analyses. The LppOmpA fusions with Chitinase A constituted either a relatively homogeneous population of medium-expressers (Fig. 4a, left panel), or a slightly more heterogeneous population with some very highly expressing cells (Fig. 4a, right panel). This demonstrates how the evaluation of production efficiency may vary dependent on the read-out method, and how the versatility of the NB:GFP platform allows for optimisation of several important parameters. This will likely be of high value for development of robust whole-cell catalysts. The occurrence of two populations can in the context of the present study be attributed to the rhamnose promoter: when expressing the LppOmpA-NB construct under control of the IPTG-inducible *P*_trc_ promoter, we observed an almost homogeneous population distribution (Additional file 2: Figure S2).

We have used the platform to evaluate one of the key parameters for secretion, the N-terminal signal sequence responsible for targeting the nascent polypeptide to the periplasm prior to its translocation across the outer membrane [50]. This demonstrated the potential of the NB:GFP platform to study the effect of different signal peptides in surface presentation, in a multi-format setup. Fluorescence signals varied more than sixfold between the lowest and highest displayed constructs. In consistence with previous reports [51], this highlights the importance of systematic process optimisation for surface display, and shows the feasibility of the developed technology. The negligibly low torA signal is not unexpected since this peptide only directs export of fully folded protein [52]. Compared to the original pK construct, all tested signal sequences gave a lower fluorescence signal, which is likely explained by the fact that pK is a high-copy plasmid whereas the signal peptide library backbone is a low-copy vector. Inserted into pK, however, gIII no longer excelled compared to pelB. This shows that surface display is dependent on an intricate mesh of mechanisms, where plasmid copy number and signal sequence are two important, interdependent parameters, making display optimisation a complex process.

The NB:GFP system is a rapid assay for quantitative assessment of surface display, making use of GFP that is easily produced and purified in *E. coli*. The platform has the potential to ease systematic studies of surface display systems and drive quick optimisation of individual display systems in a multi-well format—however, optimisation and testing of the system for each individual protein will likely be needed. Furthermore, our hope is that the NB:GFP platform will facilitate the fundamental understanding of the molecular mechanisms behind the biogenesis of *E. coli* outer membrane proteins that can enable rational development of bacterial surface display systems and robust whole-cell biocatalysts in the future.

## Conclusions

We developed an inexpensive, robust and quantitative surface display platform that allows for functional display of enzymes. Furthermore, we employed the nanobody:GFP platform for (1) expression of the industrially relevant enzyme Chitinase A and (2) evaluation of a signal peptide library’s effect on surface display through easy quantitative screening.

## Methods

### Gene and vector design

All cloning was done with USER fusion (as described in Cavaleiro et al. [53]) unless otherwise specified, into the high-copy plasmid vector pK (described in [33]) and is outlined in Additional file 4: Figure S4. C-IgAP sequence and the N-terminal signal peptide pelB were obtained from the de Lorenzo lab [19] and cloned into pK using oligos 525, 526, 527 and 528, forming pK:C-IgAP. pK:lpp-OmpA was constructed in several steps: Lpp and OmpA were amplified from *E. coli* K12 MG1655 genomic DNA with oligos Lpp-F and Lpp-OmpA-R, and OmpAR and Lpp-OmpA-F, respectively, thereby constructing the previously described LppOmpA chimera [21]. They were cloned into plasmid pGFP (described in [54]) using oligos pGFP_1 and pGFP_2, creating plasmid pLppOmpA-GFP. Later, LppOmpA-GFP was transferred to the pK vector with oligos GFPgenR and Lpp-F for LppOmpA-GFP amplification, and oligos 525 and 526 for opening pK. Subsequently, the nanobody sequence was ordered as a G-block (Genscript) codon optimized for *E. coli*, and cloned into plasmid pGFP with restriction cloning using *XhoI* and *HindIII* sites. The nanobody was then cloned C-terminally to LppOmpA, replacing GFP, using oligos 855, 856, 857 and 858, creating plasmid pK:LppOmpA-NB. N-terminal fusion of the nanobody to the autotransporter C-IgAP, forming pK:NB-C-IgAP, was done using oligos 1715, 1716, 1717, and 1718. The Chitinase A gene was provided by the de Lorenzo lab, and was inserted C- or N-terminally to LppOmpA by cloning with oligos 1889, 1890, 1891, 1892, 1898, and 1899. pK_ChiA-NB-AT was constructed by cloning with oligos 1889, 1895, 1898, and 1899. All oligos and plasmids can be found in Additional file 5: Table S1 and Additional file 6: Table S2, respectively.

### Bacterial strains and culture conditions

*Escherichia coli* NEB5alpha strain (New England Biolabs) was used for cloning purposes and *E. coli* OneShot BL21(DE3) (ThermoScientific) were used for protein production and display. All cultures were grown at 37 °C in Luria Bertoni broth (LB) under agitation, unless otherwise noted, with kanamycin supplemented to 50 µg/ml to maintain the pK plasmids.

### Production of surface displayed proteins

Surface display ORFs were under the control of the *rhaP*_*BAD*_ promoter. *E. coli* BL21(DE3) cells containing the pK surface display plasmids were inoculated from overnight culture to OD_600_ 0.1 in LB and grown at 37 °C, 250–300 rpm, to an OD_600_ of 0.3–0.5, when protein production was induced. Induction was carried out using various concentration of rhamnose and expression of plasmid-encoded genes was allowed for 3 h at 30 °C.

### GFP and NB production and purification

His-tagged nanobody and Folding reporter GFP [55] under control of the T7 promoter were produced in *E. coli* SHuffle (New England Biolabs) and *E. coli* BL21 (DE3), respectively, by induction with 0.4 mM IPTG for 5 h. Cells were then resuspended in IMAC wash buffer (50 mM Tris–HCl, 10 mM imidazole, 500 mM NaCl, 10 % glycerol [pH 7.5]), lysed by three passes through an EmulsiFlex-C5 homogenizer (Avestin) at 10,000–15,000 psi and any debris and unbroken cells were removed by centrifuging at 18,000*g* at 4 °C for 15 min. The supernatant containing the proteins of interest was loaded onto nickel-nitrilotriacetic acid (Ni^2+^-NTA) resin columns (HisTRAP) on an Äkta Pure system connected to an F9-C fraction collector (GE). The bound protein was washed extensively with IMAC wash buffer and was subsequently eluted by increasing the imidazole concentrations to 500 mM in a single step. The fractions containing the protein of interest were pooled and stored at –80 °C for nanobody, and –20 °C for GFP, until use.

### Nanobody:GFP assay

Protein production was stopped by pelleting cells via centrifugation for 4 min at 2272*g* in a ThermoScientific Multifuge X3 FR centrifuge. Cells were then resuspended in 50 µl 50 mM Tris buffer, and mixed with 50 µl 0.12 mg/ml GFP (final concentration 0.06 mg/ml). Cells and GFP were incubated for 20 min at 30 °C at 250–300 rpm. Incubation was stopped by centrifugation of cells, 4 min at 2272*g*. Cells were washed twice with 300 µl 50 mM Tris buffer, and then resuspended in 220 µl 50 mM Tris buffer before downstream analyses.

### GFP signal detection and OD measurement using plate reader

200 µl cell suspension was transferred to an opaque microtiter plate (Sigma-Aldrich) and GFP signal was read in a SynergyMx plate reader (BioTek) at gain 80. 40 µl cells were then transferred to 160 µl 50 mM Tris buffer (1/5x dilution) in a transparent microplate (Greiner Bio-One) and optical density at 600 nm was measured.

### SDS-PAGE analysis

Cells were resuspended to a concentration of 0.05 ODU/µl and 10 µl were mixed with 5 µl 2× Laemmli sample buffer and 0.5 µl benzonase nuclease (≥250 units/µl, Sigma), after which the whole sample was loaded onto a 4–20 % Mini-PROTEAN^®^ TGX™ gel (Bio-Rad) and run for 35 min at 150 V. Fluorescent protein bands were visualised with the G:Box bioimager (Syngene) using UV-light filter, and total protein was assessed by staining with InstantBlue (Expedeon).

### Fluorescence microscopy

3 µl cells were pipetted onto Poly-prep microscopy slides (Sigma) and studied in a Leica DM4000B fluorescence microscope at 100× magnification, using Leica Application Suite v4.0 for capturing images. The GFP fluorophore was excited and signal detected using an excitation filter with band-pass 470/40 and a suppression filter with band-pass 525/50.

### Proteinase K accessibility assay

Cells were harvested and resuspended in 50 µl PBS buffer before adding 1.5 µl Proteinase K (final concentration 0.58 mg/ml) (ThermoScientific). Samples were incubated for 30 min at 37 °C, after which the accessibility was assessed by carrying out the NB:GFP assay and analysing samples on SDS-PAGE as described above, starting with a centrifugation and washing step to remove all cleaved off protein.

### Flow cytometry

Flow cytometry measurements were performed on a FACS Aria (Becton–Dickinson, San Jose, USA) with 488 nm excitation from a blue solid-state laser. Cells were diluted 1:100 in PBS for analysis. At least 20,000 cells were collected for each measurement. FlowJo (Treestar) was used for data analysis.

### Chitinase activity assay

Chitinase activity was analysed using the Chitinase Assay Kit (Sigma-Aldrich) and carried out according to the manufacturer’s instructions. Briefly, cells induced for chitinase production during 4 h were resuspended to 0.0044 OD_600_ units/µl and 10 µl (0.044 OD_600_ units) were mixed with 10 µl 4-Nitrophenyl N,N’-diacetyl-β-d-chitobioside (1 mg/ml). Samples were incubated at 37 °C for 30 min, and the reaction was stopped by addition of 200 µl 39 mM sodium carbonate solution. Cells were harvested, and 200 µl supernatant was transferred to a microtiter plate (Greiner Bio-One) and the concentration of *p*-nitrophenol was measured as absorbance at 405 nm in a SynergyMx plate reader (BioTek). Specific activity was calculated as absorption per time per volume against a *p*-nitrophenol standard.

### Cloning of *P*_trc_ construct

The open reading frame from plasmid pK:LppOmpA-NB was amplified using primers 2148 and 2152 and USER cloned into the linearized backbone pCDF_sl3m:Ptrc-GFP made from amplifying plasmid pCDF_sl3m:Ptrc-GFP [56] with oligos 2155 and 2197 using the high-fidelity polymerase Phusion U (ThermoScientific). Expression and detection was carried out as described in the methods section apart from: (1) using 1 mM IPTG as final inducer concentration instead of L-Rhamnose and (2) supplementing LB growth medium with a final concentration of 50 µg/ml spectinomycin instead of kanamycin.

### Signal sequence library cloning

A panel of signal sequences (dsbA, gIII, ompA, ompC, ompT, pelB, sufI, torA, torT) in linearized vector pD881 was acquired from DNA2.0. Cloning of surface display constructs was done in close agreement with manufacturer’s instructions by treatment with *LguI* and T4 DNA Ligase (Thermo Scientific) in Tango buffer supplemented with ATP (Thermo Scientific) prior to transformation into NEB5alpha (New England Biolabs). Oligos 2236 and 2334 were used for ChiA-NB-C-IgAP library and oligos 2235 and 2539 for the LppOmpA-ChiA-NB ditto. The gIII signal sequence was subsequently cloned to replace the pelB peptide in pK:ChiA-NB-C-IgAP using oligos 2647 and 2648.

## Additional files

10.1186/s12934-016-0474-y Cells expressing the NB-C-IgAP construct are negatively affected by induction of protein production. When induced with rhamnose of varying concentration, optical density of NB-C-IgAP cultures is decreasing drastically, and to a similar level independent of inducer concentration. LppOmpA-NB-producing cultures are also affected by induction, but less dramatically and in a stepwise manner. Values are normalised to the average of uninduced cells, biological triplicates, standard error.
10.1186/s12934-016-0474-y Population distribution with the *P*_trc_ promoter. The LppOmpA-NB fusion was cloned into a vector containing the IPTG-inducible *P*_trc_ promoter and subsequently assayed according to the described NB:GFP procedure, followed by flow cytometry analysis. As depicted in the figure, a large majority of cells were fluorescent.
10.1186/s12934-016-0474-y Variation in display signal when fusing OmpA-ChiA-NB to a set of 9 different signal peptides. The OmpA-ChiA-NB fusion protein was directed to the cell surface by different signal peptides, leading to varying levels of surface display. Values are averages of three biological replicates, error bars standard errors.
10.1186/s12934-016-0474-y Overview of plasmid construction. Oligos are given in bold font, plasmid names in regular font under the plasmid. Colours show from which source each fragment is amplified. The pK:LppOmpA-NB plasmid was made in several steps, starting with amplification of LppOmpA from the *E. coli* chromosome, whereas pK:C-IgAP was created in one step. The nanobody sequence was ordered as a G-block and restriction cloned into pGFP. Plasmids with Chitinase A were made by cloning of the ChiA gene into pK:LppOmpA-NB and pK:NB-C-IgAP. Details are found in Gene and Vector design in the Methods section.
10.1186/s12934-016-0474-y Oligos used in this study.
10.1186/s12934-016-0474-y Plasmids used in this study.

## Abbreviations

NB: nanobody
GFP: green fluorescent protein
OmpA: outer membrane protein A
C-IgAP: C-terminal part of the IgA protease of *Neisseria gonorrhoea*
OD: optical density
