# Supplementary figures and images for: A nanobody:GFP bacterial platform that enables functional enzyme display and easy quantification of display capacity
Source: Microb Cell Fact. 2016 May 3;15:71. doi: 10.1186/s12934-016-0474-y (PMC4855350; doi:10.1186/s12934-016-0474-y)

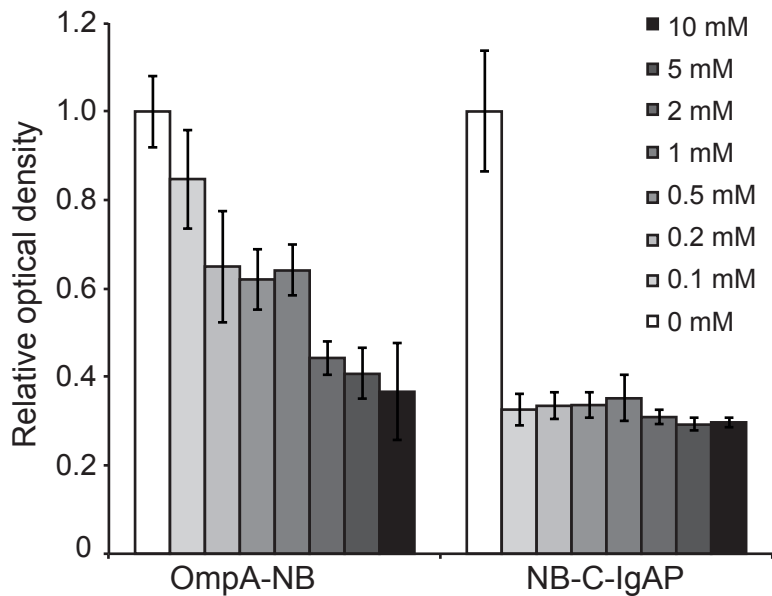

Supplement: Supplementary file 1 — 10.1186/s12934-016-0474-y Cells expressing the NB-C-IgAP construct are negatively affected by induction of protein production. When induced with rhamnose of varying concentration, optical density of NB-C-IgAP cultures is decreasing drastically, and to a similar level independent of inducer concentration. LppOmpA-NB-producing cultures are also affected by induction, but less dramatically and in a stepwise manner. Values are normalised to the average of uninduced cells, biological triplicates, standard error. [file 12934_2016_474_MOESM1_ESM.pdf]

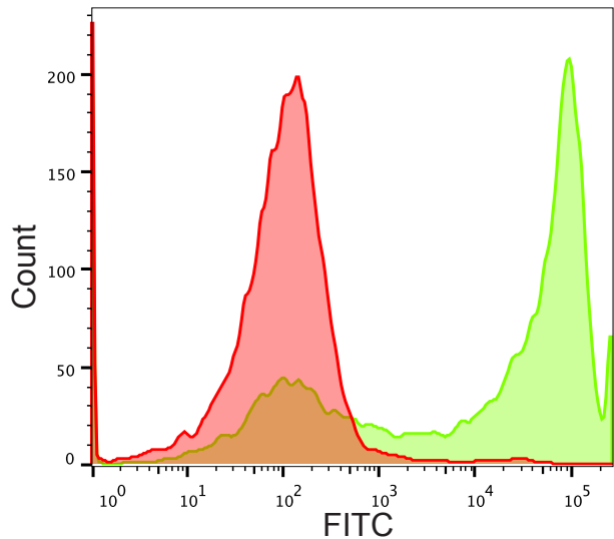

Supplement: Supplementary file 2 — 10.1186/s12934-016-0474-y Population distribution with the P trc promoter. The LppOmpA-NB fusion was cloned into a vector containing the IPTG-inducible P trc promoter and subsequently assayed according to the described NB:GFP procedure, followed by flow cytometry analysis. As depicted in the figure, a large majority of cells were fluorescent. [file 12934_2016_474_MOESM2_ESM.pdf]

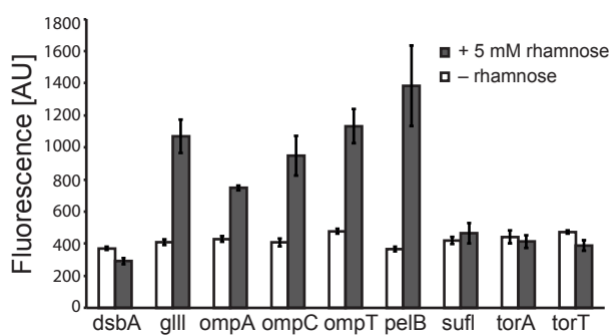

Supplement: Supplementary file 3 — 10.1186/s12934-016-0474-y Variation in display signal when fusing OmpA-ChiA-NB to a set of 9 different signal peptides. The OmpA-ChiA-NB fusion protein was directed to the cell surface by different signal peptides, leading to varying levels of surface display. Values are averages of three biological replicates, error bars standard errors. [file 12934_2016_474_MOESM3_ESM.pdf]

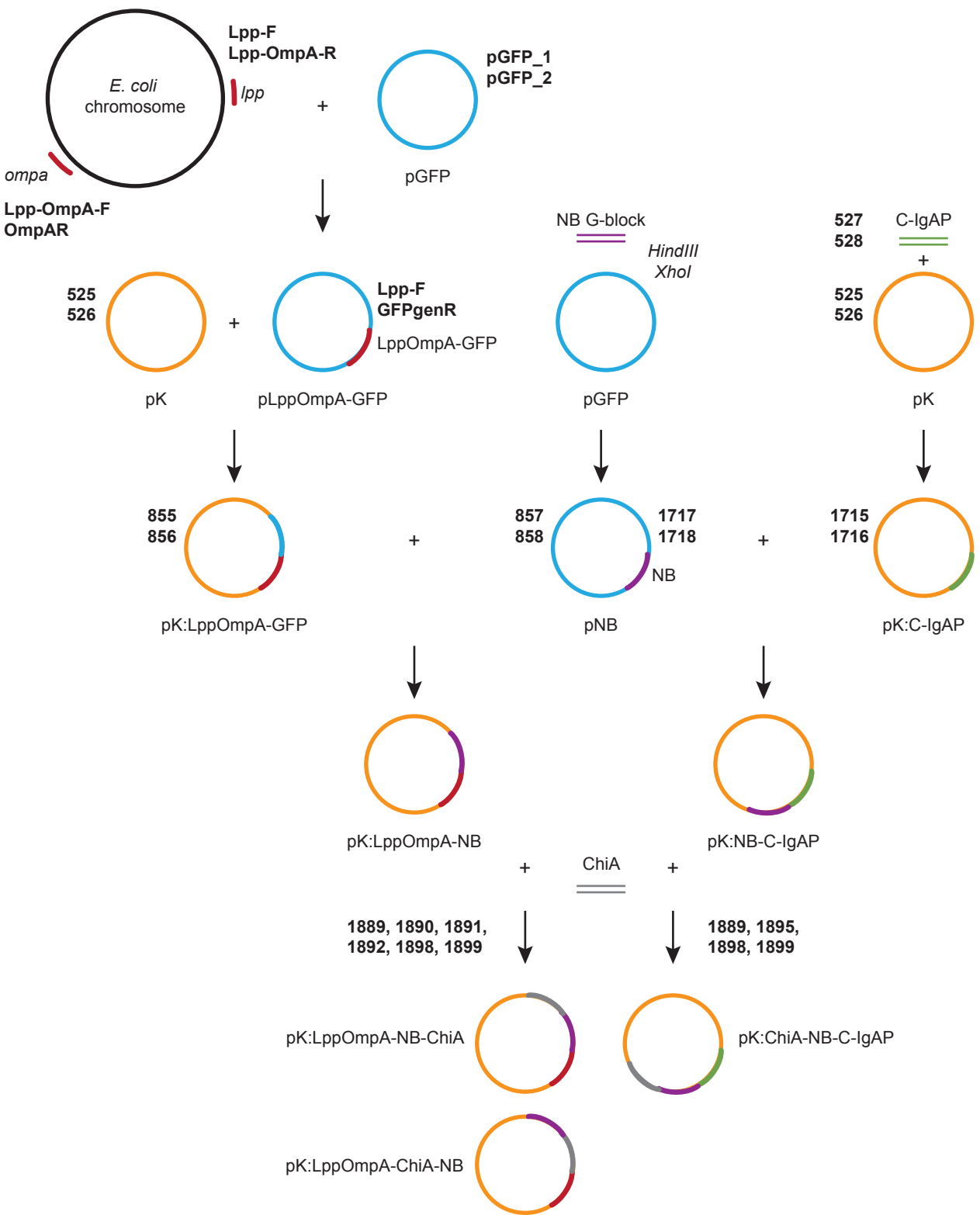

Supplement: Supplementary file 4 — 10.1186/s12934-016-0474-y Overview of plasmid construction. Oligos are given in bold font, plasmid names in regular font under the plasmid. Colours show from which source each fragment is amplified. The pK:LppOmpA-NB plasmid was made in several steps, starting with amplification of LppOmpA from the E. coli chromosome, whereas pK:C-IgAP was created in one step. The nanobody sequence was ordered as a G-block and restriction cloned into pGFP. Plasmids with Chitinase A were made by cloning of the ChiA gene into pK:LppOmpA-NB and pK:NB-C-IgAP. Details are found in Gene and Vector design in the Methods section. [file 12934_2016_474_MOESM4_ESM.pdf]
