# Supplementary material for: A nanobody:GFP bacterial platform that enables functional enzyme display and easy quantification of display capacity
Source: Microb Cell Fact. 2016 May 3;15:71. doi: 10.1186/s12934-016-0474-y (PMC4855350; doi:10.1186/s12934-016-0474-y)
Supplement: Supplementary file 5 — 10.1186/s12934-016-0474-y Oligos used in this study. [file 12934_2016_474_MOESM5_ESM.pdf]

Table S1. Oligos used in this study

Oligo ID	Sequence (5' to 3')
Lpp-F	ATATACCAUGAAAGCTACTAAACTGGTACTGG
Lpp-OmpA-R	aattccCUGATCGATTTTAGCGTTGCTGGAG
OmpAR	ACCCGGACCUCCGTTGTCCGGACGAGTGCCGATG
Lpp-OmpA-F	AGggaatUAACCCGTATGTTGGCTTTGAAATGGGTTAC
pGFP_1	AGGTCCGGGUATGAGCAAAGGAGAAGAACTTTTCAC
pGFP_2	ATGGTATAUCTCCTTCTTAAAGTTAAACAAAATTATTTCTAG
GFPgenR	ATCCTGGCUATTTGTAGAGCTCATCCATGCCATG
525	ATGGTATAUTCCTCCTGAATTTCATTACGAC
526	AGCCAGGAUAGAGTCGACCTGCAGGCATG
527	ATATACCAUGAAATACCTATTGCCTACGGCAG
528	ATCCTGGCUAGAAACGAATCTGTATTTTAATTTGTCCGGATTTTTG
855	ATaCCCGGACCUCCGTTGTCCGGACGAGTG
856	AGCCAGGAUAGAGTCGACCTGCAGGCATG
857	AGGTCCGGGtAUGGCTCAGGTCCAACTGGTCG
858	ATCCTGGCUAGTGGTGGTGGTGGTGATGATG
1715	AGCAAGCTUGCGGCCCCACGTCCACCAACACCG
1716	AGCCATATUTGCCATACTAATTGCGGCT
1717	AATATGGCUCAGGTCCAACTGGTCGAATC
1718	AAGCTTGCUGCTAACCGTGACCTGCGT
1889	AGTCCAGGCAGUATGGCTCAGGTCCAACTGGTCGAATC
1890	accaggaccgcuTCCGTTGTCCGGACGAG
1891	AGTCCAGGCAGUtgaAGCCAGGATAGAGTCGACCTG
1892	accaggaccgcuGGCCGCAAGCTTGCTG
1895	accaggaccgcuAATTGCGGCTGAATTGTCGATCGG
1898	agcggtcctgguATGGCCGCGCCGGGCAAG
1899	actgcctggacuTTGAACGCCGGCCAGGCTGG
2148	ATATACCuATACCATGAAAGCTACTAAACTGG
2152	ACCTCAGCutcaGGCCGCAAGCTTGCT
2155	AGGTATAuCTCCTTCTTAAAGTTAAAC
2197	AGCTGAGGuCGCCTCAGC
2235	TAGGTACGAACTCGATTGACGgctcttctaccTCAGGCCGCAAGCTTGCTG
2236	TAGGTACGAACTCGATTGACGgctcttctaccCTATCCTGGCTAGAAACGAATCTG
2334	TACACGTACTTAGTCGCTGAAgctcttctatgCATCACCATCACCATCACGCG
2539	TACACGTACTTAGTCGCTGAAgctcttctatgaTTAACCCGTATGTTGGCTTTGAA
2647	ATTCCGCuGGTGGTGCCGTTCTATAGCCATAGCATGCATCACCATCACCATCACGC
2648	AGCGGAAuCGCGAACAGCAGTTTTTCATGGTATATTCCTCCTGAATTTC
