# Supplementary material for: A nanobody:GFP bacterial platform that enables functional enzyme display and easy quantification of display capacity
Source: Microb Cell Fact. 2016 May 3;15:71. doi: 10.1186/s12934-016-0474-y (PMC4855350; doi:10.1186/s12934-016-0474-y)
Supplement: Supplementary file 6 — 10.1186/s12934-016-0474-y Plasmids used in this study. [file 12934_2016_474_MOESM6_ESM.pdf]

Table S2. Plasmids used in this study

Plasmid	Reference
pK	[33]
pK:C-IgAP	This study
pK:LppOmpA-GFP	This study
pGFP	[54]
pKa:LppOmpA-NB	This study
pKa:NB-C-IgAP	This study
pKa:Lpp-OmpA-NB-chiA	This study
pKa:LppOmpA-chiA-NB	This study
pKa:chiA-NB-C-IgAP	This study
pCDF_sl3m:Ptrc	[56]
pCDF_sl3m:Ptrc-LppOmpA-NB	This study
pD881	DNA2.0
pD881:dsbAss-ChiA-NB-AT	This study
pD881:gIIIss-ChiA-NB-AT	This study
pD881:ompAss-ChiA-NB-AT	This study
pD881:ompCss-ChiA-NB-AT	This study
pD881:ompTss-ChiA-NB-AT	This study
pD881:pelBss-ChiA-NB-AT	This study
pD881:sufIss-ChiA-NB-AT	This study
pD881:torAss-ChiA-NB-AT	This study
pD881:torTss-ChiA-NB-AT	This study
pD881:dsbAss-OmpA-ChiA-NB	This study
pD881:gIIIss-OmpA-ChiA-NB	This study
pD881:ompAss-OmpA-ChiA-NB	This study
pD881:ompCss-OmpA-ChiA-NB	This study
pD881:ompTss-OmpA-ChiA-NB	This study
pD881:pelBss-OmpA-ChiA-NB	This study
pD881:sufIss-OmpA-ChiA-NB	This study
pD881:torAss-OmpA-ChiA-NB	This study
pD881:TorT-OmpA-ChiA-NB	This study
pKa_gIII_chi-NB-AT	This study
